# Clinical and Functional Study of a De Novo Variant in the PVP Motif of Kv1.1 Channel Associated with Epilepsy, Developmental Delay and Ataxia

**DOI:** 10.3390/ijms23158079

**Published:** 2022-07-22

**Authors:** Giorgia Dinoi, Michael Morin, Elena Conte, Hagar Mor Shaked, Maria Antonietta Coppola, Maria Cristina D’Adamo, Orly Elpeleg, Antonella Liantonio, Inbar Hartmann, Annamaria De Luca, Rikard Blunck, Angelo Russo, Paola Imbrici

**Affiliations:** 1 Department of Pharmacy-Drug Sciences, University of Bari “Aldo Moro”, 70125 Bari, Italy; giorgia.dinoi@uniba.it (G.D.); elena.conte@uniba.it (E.C.); maria.coppola@uniba.it (M.A.C.); antonella.liantonio@uniba.it (A.L.); annamaria.deluca@uniba.it (A.D.L.); 2 Department of Physics, Université de Montréal, Montreal, QC H3C 3J7, Canada; michael.morin.1@umontreal.ca (M.M.); rikard.blunck@umontreal.ca (R.B.); 3 CIRCA, Center for Interdisciplinary Research on Brain and Learning, Université de Montréal, Montreal, QC H3C 3J7, Canada; 4 Department of Genetics, Hadassah Medical Center, Jerusalem 91120, Israel; hagarmor@hadassah.org.il (H.M.S.); elpeleg@hadassah.org.il (O.E.); 5 Department of Medicine and Surgery, LUM University, 70010 Casamassima, Italy; dadamo@lum.it; 6 Pediatric Neurology Clinic, Shamir Medical Center (Assaf Harofeh), Zerifin 7033001, Israel; inbarh@shamir.gov.il; 7 Department of Pharmacology and Physiology, Université de Montréal, Montreal, QC H3C 3J7, Canada; 8 Child Neurology Unit, IRCCS, Institute of Neurological Sciences of Bologna, 40139 Bologna, Italy; russo.neuroped@gmail.com

**Keywords:** *KCNA1*, ataxia, epilepsy, PVP motif, patch clamp, molecular dynamics, lacosamide

## Abstract

Mutations in the *KCNA1* gene, encoding the voltage-gated potassium channel Kv1.1, have been associated with a spectrum of neurological phenotypes, including episodic ataxia type 1 and developmental and epileptic encephalopathy. We have recently identified a de novo variant in *KCNA1* in the highly conserved Pro-Val-Pro motif within the pore of the Kv1.1 channel in a girl affected by early onset epilepsy, ataxia and developmental delay. Other mutations causing severe epilepsy are located in Kv1.1 pore domain. The patient was initially treated with a combination of antiepileptic drugs with limited benefit. Finally, seizures and ataxia control were achieved with lacosamide and acetazolamide. The aim of this study was to functionally characterize Kv1.1 mutant channel to provide a genotype–phenotype correlation and discuss therapeutic options for *KCNA1*-related epilepsy. To this aim, we transfected HEK 293 cells with Kv1.1 or P403A cDNAs and recorded potassium currents through whole-cell patch-clamp. P403A channels showed smaller potassium currents, voltage-dependent activation shifted by +30 mV towards positive potentials and slower kinetics of activation compared with Kv1.1 wild-type. Heteromeric Kv1.1+P403A channels, resembling the condition of the heterozygous patient, confirmed a loss-of-function biophysical phenotype. Overall, the functional characterization of P403A channels correlates with the clinical symptoms of the patient and supports the observation that mutations associated with severe epileptic phenotype cluster in a highly conserved stretch of residues in Kv1.1 pore domain. This study also strengthens the beneficial effect of acetazolamide and sodium channel blockers in *KCNA1* channelopathies.

## 1. Introduction

The voltage-gated potassium channel Kv1.1, encoded by the *KCNA1* gene, is widely distributed in the central and peripheral nervous system, where it regulates nerve cell repolarization after an action potential and contributes to neuronal excitability, firing rate and neurotransmitter release [1,2]. The channel, belonging to the Shaker-like potassium channel family, is formed by the tetrameric assembly of four Kv1.1 alpha subunits, each comprising six transmembrane segments that include functionally critical voltage-sensing (S1–S4) and pore domains (S5–S6) [3,4]. The *KCNA1* gene is well-known to scientists because several mutations in this gene have been associated with episodic ataxia type 1 (EA1), a rare disorder of cerebellar origin characterized by recurrent episodes of ataxia and myokymia since early childhood [5]. The improvements in clinical and genetic diagnosis have contributed to broaden the phenotypic spectrum associated with *KCNA1* mutations, which now includes seizures, cognitive impairment, migraine, paroxysmal kinesigenic dyskinesia, hypomagnesemia, metabolic alterations and cataplexy [6,7]. Interestingly, EA1 patients carrying *KCNA1* mutations show increased incidence of epilepsy and, in the last years, variants in *KCNA1* have been associated with epilepsy phenotypes with or without ataxia [8,9,10,11,12,13] (Table S1). This is not surprising if one considers that *kcna1* knock out mice have increased seizures susceptibility and undergo premature death (SUDEP) [14]. Since 2018, at least four mutations in the pore domain of Kv1.1 channel have been described in patients with early onset developmental and epileptic encephalopathy (DEE) [15,16,17,18]. Three of these mutations alter the two proline residues in the Pro-Val-Pro (PVP) motif at the C-terminal end of S6 (P403S, P405S, P405L), whereas the fourth is located close to the selectivity filter for potassium ions in the S5–S6 loop (V368L). The PVP region is highly conserved amongst the voltage-gated K+ channels and has been shown to be crucial for S6 flexibility and channel gating [19,20]. At the corresponding positions in *KCNA2*, the de novo P405L and P407A mutations have been associated with neurodevelopmental syndromes in which epilepsy, motor and cognitive delay co-exist [21].

Here, we report another de novo missense variant, P403A, in the PVP motif of the Kv1.1 channel in a 5-year-old girl who presents epilepsy, developmental delay and ataxia. Through patch-clamp electrophysiology and molecular dynamics simulations, we elucidated the impact of the amino acid change on channel gating and carried out a review of the literature to gain insight into the genotype–phenotype drug response correlation of nearby mutations. Our study supports previous findings, indicating that the functional impairment of residues within the pore domain result in more severe phenotypes in which epilepsy coexist with developmental delay. We also suggest the need for a careful revision of the clinical phenotype and of the therapeutic management of patients carrying *KCNA1* mutations to personalize the treatment and improve the therapeutic outcome.

## 2. Results

### 2.1. Clinical Diagnosis and Follow Up

The patient is a 5-year-2-month-old female who was born full term via spontaneous vaginal delivery after an uncomplicated pregnancy. Since previous pregnancy was terminated due to fetal tracheo-esophageal fistula, a chromosomal microarray analysis obtained by amniocentesis was performed, resulting negative.

**Figure 1:**
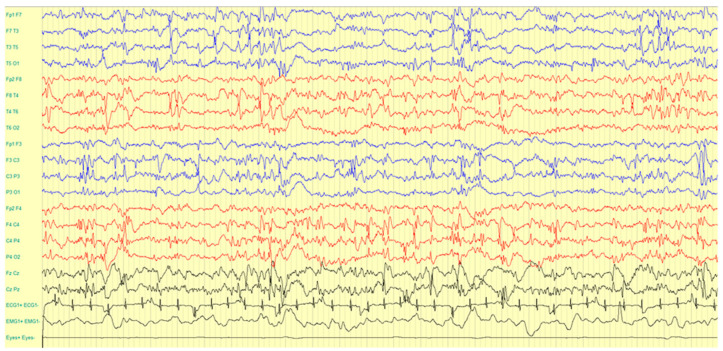
EEG during NREM sleep showing frequent and independent epileptiform abnormalities over bilateral frontotemporal regions.

At 5 days of life, she presented with a sudden onset of many seizures per week, characterized by staring. EEG showed frequent and independent medium–high amplitude sharp waves over bilateral frontotemporal regions, activated during NREM sleep (Figure 1).

Brain MRI, lumbosacral and renal ultrasound, echocardiogram, ophthalmologic examination and metabolic screening (blood lactate, amino acids, ammonia, urine organic acids, cerebrospinal fluid analysis for amino acids, glucose and lactate, very long chain fatty acid, neurotransmitters, blood and cerebrospinal fluid cultures) were normal. No dysmorphic features were present. Neurodevelopmental delay was noticed in the months following the seizure onset. At 13–15 months of age she presented other neurological signs, including eye blinking, ataxia and intentional tremor. Seizures were never controlled by antiepileptic treatment, despite attempts with several antiepileptic drugs (AEDs), such as levetiracetam, sulthiame, topiramate, valproate, cannabidiol, oxcarbazepine and clobazam. Moreover, a ketogenic diet was tried without benefits. Follow-up EEGs showed normal background activity with independent medium–high amplitude sharp wave over bilateral centrotemporal regions, with variable activation of these abnormalities during NREM sleep, but without configuring a continuous spike and wave pattern.

Seizures freedom and improvement in motor function were obtained when acetazolamide was added to lacosamide.

At last follow-up (5 years of age), she was able to walk using a hard walker and presented with intellectual disability, with an intentional distal tremor at the upper limbs and ataxia.

### 2.2. Genetic Diagnosis

Trio exome analysis of the index patient yielded 43 million mapped reads with a mean coverage of 60×. Following alignment to the reference genome (hg19) and variant calling, we performed a series of filtering steps. These included removing variants that were called less than ×8, were off-target, synonymous or had MAF  >  0.1% at gnomAD v2.1.1. There were no homozygous variants or pairs of variants in the same gene, within the exons of CGD genes among them. Compared with the parents’ samples, a heterozygous non-inherited variant was found: Chr12: 5021751 C > G, NM_000217.2: c.1207C > G, P403A, in the *KCNA1* gene. This variant does not appear in large exome databases (gnomAD), the position is preserved, and the change is predicted to be pathogenic. In addition, a variant in a homologous position of the *KCNA2* gene (P405L) has been previously described as pathogenic [22].

### 2.3. Functional and Molecular Characterization of Kv1.1P403A Channels

**Figure 2:**
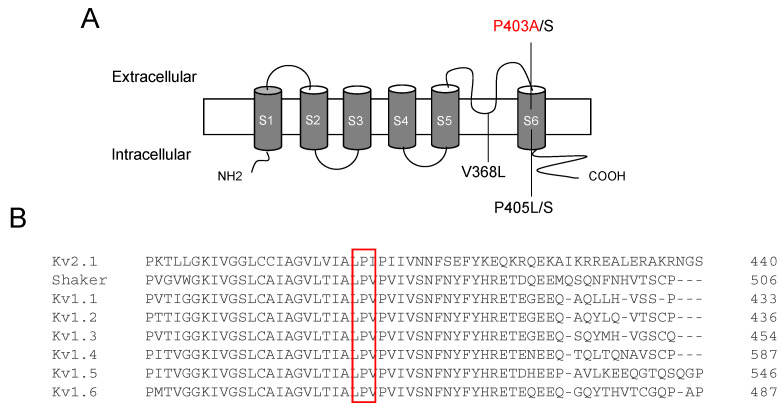
(**A**) Position of identified mutations in the Kv1.1 channel S5–S6 regions, including the P403A variant. (**B**) Amino acid alignment of Kv1 channels.

The variant P403A is located in the PVP motif in the middle of the S6 segment of the Kv1.1 channel (Figure 2A). This residue is highly conserved within the members of the Kv1 family (Figure 2B).

**Figure 3:**
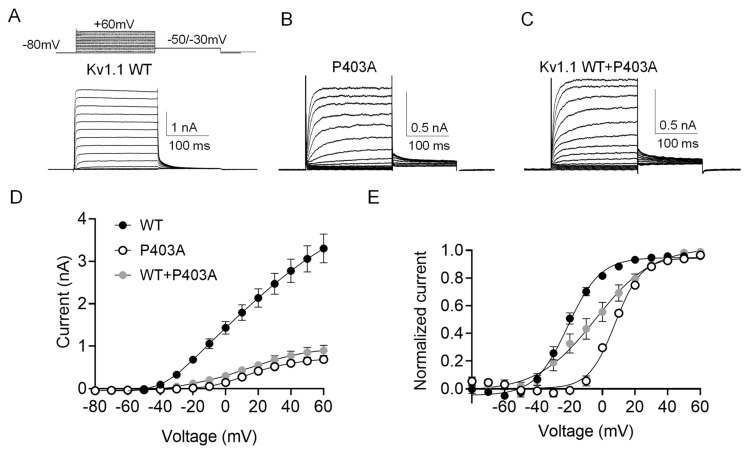
(**A**–**C**) Representative current traces evoked by 200 ms depolarizing steps from a holding potential of −80 to +60 mV from Kv1.1 WT (**A**), P403A (**B**), and Kv1.1 WT+P403A (**C**) channels expressed in HEK 293 cells. The voltage protocol is indicated in the upper panel in (**A**). (**D**) Current–voltage relationship for Kv1.1 WT (5 μg), P403A (5 μg) and Kv1.1 WT+P403A (5 μg + 5 μg) channels (n = 14–36). (**E**) Voltage-dependent activation curves for Kv1.1 WT, P403A, and Kv1.1 WT+P403A channels were obtained by plotting the normalized peak tail currents measured at −50 or −30 mV as a function of the prepulse potentials and fitting data points with a Boltzmann function (n = 10–18 cells).

As the patient is heterozygous for the mutation, Kv1.1 channels will likely be composed of Kv1.1 wild-type (WT) and P403A mutant subunits. To test the hypothesis that the P403A variant altered Kv1.1 function and caused epilepsy and ataxia in the affected patient, we transfected HEK 293 cells with equal amount of WT (5 μg) or P403A (5 μg) cDNAs alone or in 1:1 ratio (5 μg + 5 μg). As shown in Figure 3A–C, transfected HEK cells displayed typical fast activating non-inactivating potassium currents. The current amplitude and biophysical properties of potassium currents elicited by P403A and Kv1.1 WT+P403A channels were then compared with those obtained from WT currents. As shown in Figure 3D, P403A channels generate significantly smaller potassium currents at every tested potential compared with Kv1.1 WT channels (0.4 ± 0.04 vs. 2.1 ± 0.2 at + 20 mV for P403A and WT, respectively). The co-expression of Kv1.1 WT and P403A subunits gave rise to potassium currents that were less than half of the currents expected for WT channels alone (0.55 ± 0.08 at + 20 mV), suggesting a dominant-negative effect exerted by the mutant subunit on the Kv1.1 WT (Figure 3D; Table 1).

To determine whether the P403A variant may affect the voltage dependent activation, tail currents were recorded at −30 mV (for P403A and Kv1.1 WT+P403A) or −50 mV (for Kv 1.1 WT) following prepulse commands to several voltages (Figure 3E), and data points were fitted with a Boltzmann function. Mutant P403A channels displayed voltage-dependent activation significantly shifted by ~ 30 mV toward positive potentials compared to Kv1.1 WT, an effect that predicts loss-of-function (LoF) (Table 1). Potassium currents resulting from the co-transfection of WT and P403A showed voltage-dependent gating that falls between that of Kv1.1 WT and P403A homomeric channels, with a ~17 mV depolarizing shift of V_1/2_ compared to Kv1.1 WT. Conversely, the slope factor, k, calculated from the Boltzmann fit of tail currents was almost unaffected by the mutation (Figure 3E, Table 1).

**Figure 4:**
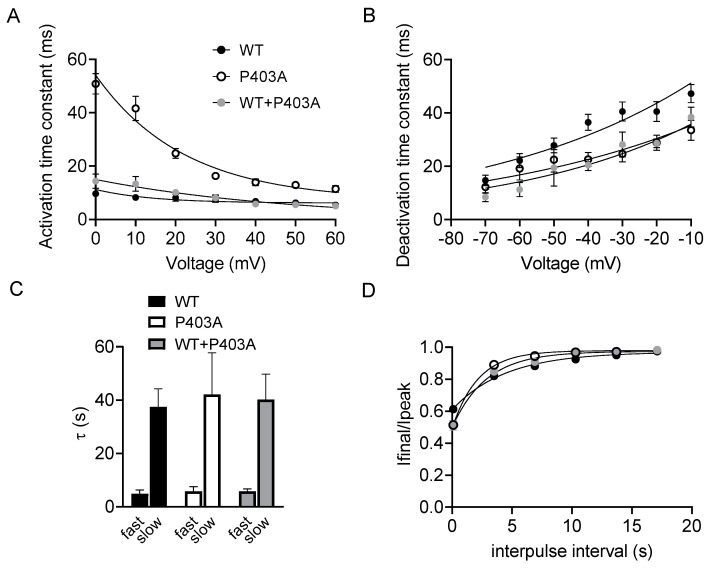
Kinetics of activation (**A**) and deactivation (**B**) measured for Kv1.1 WT, P403A and Kv1.1 WT+P403A channels. The time constants, resulting from the fit of the activating and deactivating current traces with a single exponential function, were plotted as a function of voltage (n = 10–19 cells). (**C**) Bar graphs showing the time constants of the C-type inactivation for the indicated channels calculated by fitting current decay with a single exponential function (n = 9–11 cells). (**D**) Recovery from inactivation for the indicated channels.

To investigate if the P403A variant affected the kinetics of activation and deactivation of Kv1.1 channels, the activating and deactivating current traces of either Kv1.1 WT, P403A or Kv1.1 WT+P403A channels were fitted with a single-exponential function and the calculated time constants were plotted as a function of membrane potential. The kinetics of activation of P403A channels were slower than those of Kv1.1 WT at each tested potential, indicating a LoF effect. P403A channels had kinetics of deactivation comparable with those of Kv1.1 WT (Figure 4A,B, Table 1). No differences were observed in the C-type inactivation of the three channel types (Figure 4C, Table 1). However, P403A and Kv1.1 WT+P403A recovered slightly faster from the C-type inactivation with respect to Kv1.1 WT channels (Figure 4D, Table 1).

**Figure 5:**
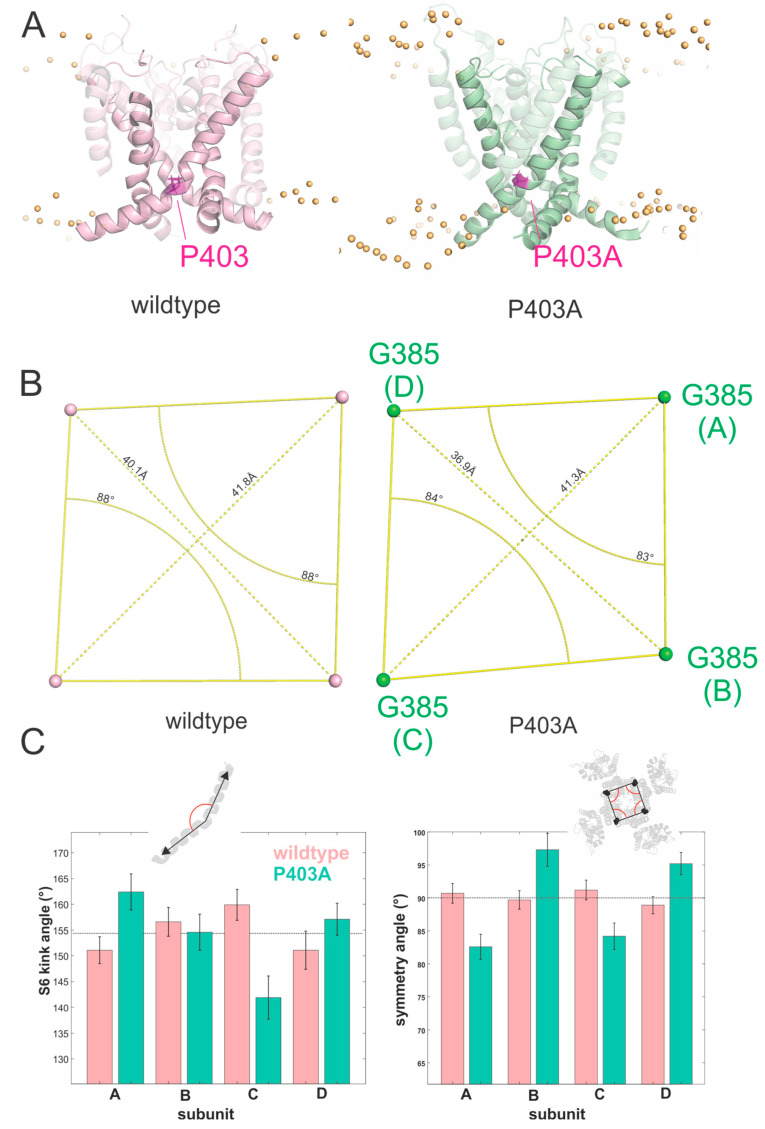
(**A**) Side view of the homology model of the Kv1.1 channel built upon the crystal structure of Kv1.2/2.1 chimera showing the localization of the P403 residue and P403A mutation. P403A is highlighted in magenta. (**B**) Loss of symmetry of wildtype and P403A measured at the top of S6 (position G385). (**C**) Measure of the S6 kink angle (*left*) and symmetry angle (*right*) for the four subunits of WT and P403A channels. The kink angle of the S6 was measured as the angle between the helix axis of the S6 above and below the PVP/A-motif. The symmetry angles are formed by the vectors from G385 of one subunit to the G385 of the two neighboring subunits. Values are given as mean ± SD of the values measured every 0.1 ns over the last 50 ns.

To understand the structural basis and molecular mechanism of the P403A mutation in Kv1.1, we created a Kv1.1 homology model based on the Kv1.2/2.1 crystal structure [4,6]. We introduced the P403A mutation in all four subunits (Figure 5A; see Section 4 for details). The sixth transmembrane helix (S6), which forms the central ion conducting pore together with the pore loop and the S5, has a pronounced kink in the WT channel caused by the PVP motif. P403A changes this motif to AVP, and, accordingly, the kink is less pronounced. The angle of the S6 kink and the angle of the C-terminal part with respect to the membrane were altered (Figure 5B). However, this effect was not consistent in all four subunits but more pronounced in one subunit versus the others (Figure 5C). In accordance with this differential conformational change, the extracellular ends of the S6 are no longer symmetrical (Figure 5B,D). Simulations of the WT channel did not show this asymmetry. We should note that in order to preserve the integrity of the selectivity filter, it had to be stabilized during the simulation. This need for stabilization and the induced asymmetry suggest that the open state of the P403A mutant is not a stable conformation. A destabilized open state would push the channel towards the closed state in accordance with the LoF phenotypes described above. A rotation of the S6 C-terminal to the PVP motif has been suggested to close the ion conduction pathway in Kv channels by turning a hydrophobic residue into the permeation pathway and dewetting the pore [23,24].

Since the simulations start with the folded structure of the WT channel and the open state structure of the P403A mutant seems to be strained, it remains a possibility that the P403A channel directly folds differently than the wildtype channel.

## 3. Discussion

### 3.1. The PVP Motif of Kv1.1 Channels as a Hotspot for Mutations Associated with Severe Epilepsy

As mentioned before, genetic variants in both *KCNA1* and *KCNA2*, which disrupt the proline residues of the PVP motif, have been identified in children with severe epilepsy and comorbidities (P403S and P405S/L in Kv1.1 and P405L and P407A in Kv1.2; Table S1). In this study, we have characterized an additional mutation in the PVP motif of the Kv1.1 channel, the P403A, associated with an epileptic phenotype.

The contribution of the PVP motif to Kv1 channel gating and the role of proline-induced kinks in transmembrane helices are well known. As suggested by the 3D structure of the rat Kv1.2 channel and of the Kv1.2/2.1 (paddle) chimera [3,4] and shown in previous mutagenesis studies, pore opening is thought to involve bending of the inner S6 helices in the vicinity of glycine gating hinge and around the PVP motif [20,25,26]. The PVP motif thus acts as a helix-breaking sequence, which provides a flexible hinge that allows the lower half of the S6 to move [23,24]. Thus, replacing proline residues in the PVP region affects S6 flexibility and pore opening and leads to drastically reduced channel activity (LoF defect). Indeed, Kv1.1 P403S and P405L channels failed to generate measurable currents when expressed in CHO cells, whereas the P403A and P405S channels showed a marked depolarizing shift in voltage-dependent activation and a significant reduction in current density [18]. In addition, the P403A subunit exerts a clear dominant-negative effect on the wild-type Kv1.1 subunit, further lowering potassium flow. Similar results have been reported for the corresponding substitution P405L in Kv1.2, which has been found in several patients with severe drug resistant phenotypes. The P405L mutant shows a dramatic reduction in current density and exerts a dominant-negative effect on Kv1.2 WT channels [22]. Of note, a de novo copy number variation in *KCNA1* extending from the PVP motif to the end of the S6 helix has been identified in a 3-year-old proband with serious myoclonic epilepsy in infancy who died of SUDEP [27].

As expected, our modelling study shows a destabilization of the open state and suggests a defective folding of the P403A channel that agrees with the reduced current amplitude and with the dominant-negative effect of the mutant subunit on the WT. Similarly, a modelling study performed on Kv1.2 channel proposed that the PVP motif may also interact with the S4–S5 linker. The substitutions of P405 and P407 in Kv1.2 by disrupting the symmetry of the PVP, might impair interactions with S4–S5 during gating-related conformational changes, leading to altered channel gating [21]. Additional evidence supports the critical role of the PVP motif for potassium channel activity and suggests that this motif can be a hotspot of mutations underlying severe epileptic phenotypes. The introduction of the P407A mutation in the Shaker Kv channel generates a non-conducting mutant channel [19]. In human Kv1.5, replacing the first proline of the PVP by an alanine results in a nonfunctional channel [28]. The mutation P403A in the PXP motif of Kv4.2 channel has been associated with early onset global developmental delay [29].

Trying to draw a correlation between epilepsy phenotype and mutation localization in *KCNA1* diseases, it appears that *KCNA1* pore mutations affecting the selectivity filter or the proline residues in the PVP motif are associated with epileptic phenotypes aggravated by developmental delay and sometimes drug-resistance, whereas aminoacidic substitutions outside the pore and the PVP domain (such as A261T, S3) seem to occur in patients with milder epileptic phenotypes, usually with a favorable seizure outcome and without intellectual disability [7,30,31] (Table S1).

### 3.2. Genotype–Phenotype–Drug Response Correlation

Regardless of the position on channel structure and clinical phenotype, all the *KCNA1* mutations identified to date, except for the A261T, cause LoF of the Kv1.1 channel expressed in heterologous cells [1,18]. Therefore, a genotype–phenotype correlation is not simple for *KCNA1* mutations and additional unclear factors, besides Kv1.1 impairment and perhaps secondary to Kv1.1 LoF, may contribute to explain different clinical phenotypes, such as those seen in twins (P403S) or among carriers of the same variant [6,21]. So far, epilepsy seems less likely to occur because of the *KCNA1* mutation with respect to EA1, requiring perhaps more deleterious defects of Kv1.1 activity, and less frequent de novo modifications and recessive inheritance have been reported. The ratio of WT/mutant subunits in the tetramer, the presence of interacting proteins, the interplay of rare and common variant in the patient, the occurrence of neuron-selective disruption and the role of Kv1.1 in neurogenesis are among the factors that could impact Kv1.1 membrane expression and gating and hence contribute to neuronal network impairment and clinical heterogeneity [1,32,33].

The presentation of seizures alongside other neurological and mental health issues in *KCNA1* carriers means that accurate diagnosis and AEDs prescribing is challenging. As in other rare neurologic channelopathies, standard therapy has not yet been established and different treatment attempts need to be evaluated case by case to reach a personalized therapeutic scheme able to reduce seizure frequency and improve patient’s quality of life. Acetazolamide, a carbonic anhydrase inhibitor, is often used to treat ataxia, with moderate benefit in most patients, including the girl described here. Sodium channel blockers proved effective to restore brain excitability in both EA1 and epilepsy [18,34,35] (Table S1). In severe epilepsy cases, however, seizures may remain intractable or poorly improve after trials with combinations of AEDs, including phenobarbital, clobazam, valproate, lamotrigine, carbamazepine and phenytoin. The P405S carrier experienced severe intractable seizures [16], whereas the patient bearing P405L slightly improved only with a combination of acetazolamide, lamotrigine, valproic acid and ACTH to specifically treat status epilepticus during sleep [17]. The P403S mutation was identified in twins showing epileptic phenotypes of different severity, with one boy presenting convulsive episodes controlled by lamotrigine and the other drug-resistant seizures [16]. The recessively inherited V368L mutation was found in a patient presenting with a combination of dyskinesia, motor and intellectual disability and neonatal epileptic encephalopathy, controlled with oxcarbazepine [15]. After having tried combinations of AEDs, seizures and ataxia were finally controlled in our patient (P403A mutation) with acetazolamide and lacosamide. Lacosamide, a drug used to treat focal epilepsy as monotherapy or as an add-on therapy, differs from other sodium channel blockers as it enhances the slow inactivation of the sodium channel without affecting the fast inactivation and also acts as a carbonic anhydrase inhibitor [36], a dual mechanism that may well motivate its efficacy. Cannabis-based medical products have recently been approved to treat seizures associated with intractable Lennox–Gastaut syndrome, Dravet syndrome or tuberous sclerosis complex in patients of 1 year of age and over [37]. Cannabidiol was unsuccessful in our patient and was rapidly discontinued, as well as ketogenic diet. Overall, the comparison of the treatment regimens of patients carrying a *KCNA1* mutation confirms the need of a patient-centric approach and supports the use of sodium channel blockers, such as carbamazepine, lacosamide, lamotrigine, to improve the course of the disease [34,38]. Caution must be taken when using sodium valproate in children due to possible seizure aggravation and clonazepam and oxcarbazepine due to motor coordination disturbances [39].

In conclusion, in depth structure–function and pharmacological studies are essential to understand the consequences of pathogenic mutations on channel activity and clinical phenotype and can also be fundamental for the rational design of drugs targeting specific mutations defects. An improved knowledge of patients’ genetic background, including the presence of common variants, protein–protein interactions and brain networks functioning will be necessary to better understand the molecular mechanisms underlying *KCNA1* channelopathies and to support therapeutic decisions.

## 4. Materials and Methods

### 4.1. Clinical and Genetic Analysis

Following informed consent, exonic sequences were enriched in the DNA sample of the proband and the parents using SureSelect Human All Exon v.5 50 Mb Kit (Agilent Technologies, Santa Clara, CA, USA). Sequences were determined by HiSeq2500 (Illumina, San Diego, CA, USA) as 100 bp paired-end runs. Data analysis, including read alignment and variant calling, was performed by DNAnexus software (Palo Alto, CA, USA) using the default parameters with the human genome assembly hg19 (GRCh37) as reference.

### 4.2. Electrophysiology

Whole-cell patch-clamp experiments were performed at room temperature (~20 °C) using an Axopatch 200B amplifier and Digidata 1550B AD/DA converter (Axon Instruments, Sunnyvale, CA, USA). Currents were low-pass filtered at 2 kHz and digitized with sampling rates of 50 kHz. The bath solution contained (mM) NaCl 142, KCl 2.8, MgCl_2_ 1, CaCl_2_ 1, HEPES 10, glucose 11, pH = 7.4, whereas the pipettes were filled with solution containing (mM) NaCl 10, K-glutamate 132, MgCl_2_ 2, CaCl_2_ 0.9, EGTA 1, HEPES 10, pH = 7.4 [40]. The pipettes were pulled from borosilicate glass and had ~4 MΩ resistance.

Potassium currents were evoked by 200 ms depolarizing commands from a holding potential of −80 to +60 mV (10 mV intervals). To measure tail currents, each prepulse was followed by a 150 ms voltage step at −50 or −30 mV for Kv1.1 wild-type and P403A channels, respectively. Normalized tail currents were plotted as a function of the prepulse potential, and data points fitted with the Boltzmann function I = 1/1 + exp{−(V − V_1/2_)/k} to determine the voltage dependence of channel activation. V_1/2_, the half-maximal activation potential, and k, the slope factor, were calculated from the fit.

To measure the kinetics of activation, currents were elicited by 200 ms depolarizing pulses from a holding potential of −80 to +60 mV (10 mV intervals). To measure the kinetics of deactivation, currents were elicited by 200 ms depolarizing pulse at +20 mV, followed by 200 ms depolarizing pulses from −80 to +20 mV (10 mV intervals). The kinetics of activation and deactivation were determined by fitting activating and deactivating current traces with a single exponential function. The calculated time constants were plotted as a function of voltage and fitted with the equation τ = τ_V1/2_ exp(V−V_1/2_)/k, where τ_V1/2_ is the time constant at the V_1/2_ of the channels, and k is the slope factor for the voltage dependence of the time constants.

To determine the slow C-type inactivation, a test pulse to +40 mV was delivered for 90 s to cells expressing wild-type and mutant channels. The kinetics of inactivation were estimated by fitting the time course of current decay with a double exponential function and calculating the fast (τ_fast_) and slow (τ_slow_) time constants and the relevant amplitudes (A%).

The recovery from the C-type inactivation was determined by using a double-pulse protocol to +20 mV, separated by interpulse intervals of increasing duration (from 0.1 to 17.1 s). The peak currents evoked by the second pulse (100 ms) were divided by the first pulse (20 s), normalized and plotted as a function of the interpulse duration. Data points were fitted with an exponential function, from which the time constants were calculated.

Data were analyzed by using pCLAMP 10.3 (Axon Instruments, Sunnyvale, CA, USA) and GraphPad Software. Results were reported as mean ± SEM from n cells, and statistical analysis was performed using Student’s *t*-test, with *p* < 0.05 (*) considered as significant.

### 4.3. Homology Modeling and Molecular Dynamics Simulations

A homology model of Kv1.1 was built from the crystal structure of the Kv1.2/2.1 chimera (PDB 2R9R) [4], as described previously [6]. Residues that differed or were missing in the Kv1.2/2.1 chimera as compared with the human Kv1.1 were replaced and modeled, respectively, using Modeller 9.25 [41,42]. We introduced the mutation P403A in the four subunits in the Kv1.1 tetramer. The N- (1–147) and C-termini (420–495) were truncated. The resulting homotetrameric transmembrane domain was introduced into a POPE: POPC: POPS membrane (3:2:1; 444 lipids total) and inserted in a hexagonal box (diagonal 170 Å, height 110 Å; filled with 150 mM KCl) using CHARMM-GUI [43,44] and was equilibrated using NAMD 2.14 with the CHARMM36 force field [45]. The simulation was run for 280 ns after equilibration.

As control condition, a homotetramer of the Kv1.1 WT channel was modelled and simulated identically. For some simulations, the selectivity filter was stabilized by imposing the geometry of one carbonyl group and the position of a single potassium ion in the selectivity filter. Analysis and graphical representation were created using in-house written python and Matlab routines using the open source Pymol library.

## Figures and Tables

**Table 1:** Biophysical parameters of Kv1.1 WT, P403A and Kv1.1 WT+P403A channels expressed in HEK 293 cells.

	Voltage Dependence of Activation		Kinetics of Activation	Kinetics of Deactivation	C-Type Inactivation		Recovery from Inactivation
	V_1/2_ (mV)	k (mV)	τ_V1/2_ (ms)	τ_V1/2_ (ms)	τ_fast_ (s)	τ_slow_ (s)	τ (s)
**Kv1.1 WT**	−21.5 ± 0.4 (29)	10.0 ± 0.8 (29)	9.4 ± 0.9 (15)	41.5 ± 2.4 (16)	4.9 ± 1.4 (10)	37.4 ± 6.7 (10)	3.4 ± 0.6 (7)
**P403A**	7.8 ± 0.4 * (13)	9.0 ± 0.8 (13)	31.7 ± 6.0 * (14)	47.2 ± 2.6 (14)	5.8 ± 1.7 (7)	42.1 ± 15.7 (7)	2.2 ± 0.3 (9)
**Kv1.1 WT+P403A**	−4.3 ± 0.7 * (10)	12.0 ± 0.9 (10)	14.7 ± 3.1 (10)	41.5 ± 1.6 (7)	5.8 ± 0.9 (9)	40.2 ± 9.5 (9)	2.8 ± 0.3 (9)

Data are mean ± SE. The number of cells is indicated in brackets. * *p* < 0.05, with respect to WT.

## Data Availability

The data that support the findings of this study are available from the corresponding authors upon reasonable request.

## Supplementary Materials

The following supporting information can be downloaded at: https://www.mdpi.com/article/10.3390/ijms23158079/s1.
