# Supplementary material for: Clinical and Functional Study of a De Novo Variant in the PVP Motif of Kv1.1 Channel Associated with Epilepsy, Developmental Delay and Ataxia
Source: Int J Mol Sci. 2022 Jul 22;23(15):8079. doi: 10.3390/ijms23158079 (PMC9331732; doi:10.3390/ijms23158079)
Supplement: Supplementary file 1 [file ijms-23-08079-s001.zip › ijms-1792331-supplementary.pdf]

Table S1. *KCNA1* mutations associated with ataxia and epilepsy and with DEE

Mutation	Position	Clinical symptoms	Functional defects	Treatment	References
**F184C**	S1	Severe ataxia, severe neuromyotonia, epilepsy, visual disturbances	LoF, Reduced current density and positive shift of voltage-dependent activation	Phenytoin partially effective	[8]
**T226R**	S2	Moderate ataxia, severe neuromyotonia, epilepsy, cataplexy, sleep disturbances, skeletal deformities	LoF, Reduced current density	Acetazolamide and carbamazepine effective; phenytoin, valproic acid and phenobarbital partially effective	[7]
**A242P**	S2	Moderate ataxia, myokymia, epilepsy	LoF, Reduced current density	Lamotrigine effective; acetazolamide ineffective	[29]
**A261T (de novo)**	S3	Fever-induced seizures, ataxia, myokymia	GoF, Negative shift of voltage-dependent activation	Carbamazepine effective, valproate partially effective	[12, 18]
**L319R**	S4-S5	Paroxysmal kinesigenic dyskinesia without ataxia; in some carriers seizures	LoF, Reduced current density and dominant-negative effect, positive shift of voltage-dependent activation	Oxcarbazepine or carbamazepine effective	[9]
**R324T**	S4-S5	Moderate ataxia, severe neuromyotonia, epilepsy, suspected paroxysmal kinesigenic dyskinesia	LoF, Reduced current density	Carbamazepine effective	[10]
**S342I**	S5	Ataxia and seizures	NA	Phenytoin effective	[11]
**V368L**	S5-S6	DEE, ataxia, myokymia	LoF, Non-functional channels	Phenobarbital partially effective, valproic acid and oxcarbazepine effective	[15]
**G376S**	S5-S6	Seizures, mild cognitive and motor delay, ataxia, myokymia	NA	Valproic acid effective	[12]
**P403S (de novo) In twins**	PVP, S6	DEE; Twin A: early onset epilepsy, moderate cognitive and motor development delay; ataxia. Twin B: epilepsy, severe intellectual disability, ataxia and myokymia, language loss.	LoF, Non-functional channels	Twin A: lamotrigine partially effective. Twin B: drug-resistant seizures. Combinations of valproic acid, vigabatrin, lamotrigine, oxacarbazepine, phenytoin and clobazam poorly effective	[16]
**P403A (de novo)**	PVP, S6	Epilepsy, ataxia and intellectual disability	LoF, Reduced current density and dominant-negative effect, positive shift of voltage-	Lacosamide and acetazolamide effective	This study

			dependent activation, slower kinetics of activation		
**P405S (de novo)**	PVP, S6	DEE, cognitive impairment; fever-induced seizures.	LoF, Markedly reduced current density and marked depolarizing shift of voltage-dependent activation	Drug-resistant seizures. At last follow up treatment comprises: clobazam, carbamazepine, zonisamide and valproic acid poorly effective. Phenobarbital ineffective	[16]
**P405L (de novo)**	PVP, S6	DEE, neonatal onset epilepsy, developmental delay, status epilepticus during sleep (ESES)	LoF, Non-functional channels	Phenobarbital, carbamazepine, levetiracetam ineffective and phenitoin partially effective. At last follow up treatment comprises: acetazolamide, lamotrigine and valproic acid with seizures control. ESES responsive to ACTH	[16, 17]
**V408L**	S6	Global developmental delay, myokymia with postural abnormalities, mild ataxia, seizures	LoF, Faster inactivation	Carbamazepine or phenytoin effective	[13]
**F414C**	C-term	Moderate episodic ataxia, myokymia, neuromyotonia, isolated photosensitive generalized tonic-clonic seizure	LoF, Non-functional channels and dominant-negative effect	Clonazepam partially effective; acetazolamide and oxcarbazepine ineffective	[30]
**R417Stop**	C-term	Severe ataxia, dysarthria and slurred speech; periocular myokymia; epilepsy and tremor	LoF, Non-functional channels and dominant-negative effect	Carbamazepine and acetazolamide partially effective; lamotrigine, vigabatrin and clonazepam ineffective	[31]

NA = Not available; DEE = developmental and epileptic encephalopathy; LoF = loss-of-function
